# The ongoing evolution of mentorship: Advancing the formal mentorship program at AKU-SONAM

**DOI:** 10.12669/pjms.40.3.8212

**Published:** 2024

**Authors:** Rehana Rehman, Tazeen Saeed Ali, Saira Khalid, Rahila Ali

**Affiliations:** 1 Rehana Rehman Professor, Department of Biological & Biomedical sciences, Aga Khan University, Karachi. Pakistan; 2 Tazeen Saeed Ali Interim Dean, School of nursing and Midwifery Aga Khan University, Karachi. Pakistan; 3 Saira Khalid Nursing Instructor, College of Nursing Armed Forces Postgraduate, Medical Institute (AFPGMI), Rawalpindi. Pakistan.; 4 Rahila Ali Senior Instructor, Department for Educational Development, Aga Khan University, Karachi. Pakistan

**Keywords:** Mentorship, Mentors, Mentees, AKU-SONAM, Evolution

## Abstract

**Objective:**

To investigate the perceptions of mentors, mentees, administrators (including chairs, co-chairs, and coordinators of the mentoring program), and leadership regarding the Faculty Mentorship Program at AKU School of Nursing and Midwifery (AKU-SONAM). Additionally, the study aimed to explore the strengths and challenges faced by the program.

**Methods:**

A qualitative exploratory study on mentorship was carried out in AKU-SONAM from February till December 2021. In this study we conducted purposive criterion sampling. The study participants included mentors, mentees, administrators, and the dean of AKU-SONAM. An interview guide was developed, validated, and reviewed by experts. After piloting, two focused group discussions and three in-depth interviews were conducted.

**Results:**

Thematic analysis of the data revealed the following themes: “Nurturing growth and development in mentoring relationships,” “Establishing a strong foundation for effective mentoring,” and “Opportunities for growth and development through overcoming challenges.”

**Conclusion:**

The participants expressed confidence in the existing support system, leadership, and mentorship program at the institution. They highlighted the significance of regular monitoring to recognize areas of improvement uphold high standards and ensure excellence.

## INTRODUCTION

“Mentoring is a reciprocal and collaborative learning relationship between two, sometimes more, individuals with mutual goals and shared accountability for the outcomes and success of the relationship”.^1^ It is a supportive, nurturing and reciprocal relationship between mentees and mentors for substantial benefits to both as well as the organization.^2^

Mentorship programs have been initiated for students in medical universities of Pakistan with endorsement of mentors role in their academic productivity and emotional wellbeing.^3^ Talking about formal faculty mentorship; it provides a structured framework to support faculty members throughout their careers, promoting their success, and ultimately contribute to the overall growth and excellence of the education system.^4^,^5^ The effective mentoring relationships then lead to improved teaching performance, enhanced scholarly productivity, successful promotion and tenure outcomes.^6^

In nursing practice, mentoring can be tracked back to the Nightingale model of professional development, however transformation from informal to formal mentoring is documented to retain nurses and nurture leadership skills at their workplace.^6^ The mentorship program at AKU-School of Nursing and Midwifery (AKU-SONAM) was established in 2002 with the purpose of providing guidance, support, encouragement, and adaptation assistance to newly appointed nurse faculty, tailored to their interests, educational background, and individual needs. The program was restructured and then formalized in 2019. Recent literature on faculty mentoring in nursing schools has identified impact of mentoring on professional growth and career satisfaction.^7^ Therefore, we aimed to explore faculty Mentorship Programs at AKU-SONAM by collecting perceptions of faculty (mentor, mentee), administrators and leadership regarding existing program.

## METHODS

A qualitative exploratory study on mentorship was carried out in AKU-SONAM from February till December 2021. Purposive sampling technique was used to recruit mentors, mentees, administrators, and leadership. Mentors and mentees who had been involved in the mentorship program of AKU- SONAM for at least one year and had participated in a minimum of two mentoring sessions were included. Administrators consisted of chairs, co-chairs and coordinators of the mentorship program, with at least one year of service. The leadership category included the Dean of AKU-SONAM.

### Ethical Approval:

It was obtained from the Aga Khan University, Karachi (2021-6127-17832).

### Sample Size Calculation:

Generalizability is not considered in qualitative studies; therefore, we obtained a list of all administrators, mentors and mentees invited them for In-depth interviews (IDIs) and Focus groups (FGDs). The collection process was closed when there was repetition of responses and when no more meaningful information, new codes or categories or themes could be evolved 8.

### Data collection sources:

IDIs with leadership and administrators and FGDs were chosen for mentors and mentees. Document reviews included guidelines, policies and other relevant available records.

### Development of tool:

A semi-structured guide was developed for both IDIs and FGDs based on relevant literature.

### Validation of tool:

The guide underwent multiple rounds of review by experts experienced in mentoring and qualitative studies using the Delphi method.

### Pilot study:

After piloting the interview guides, the interviews were conducted in a private setting to ensure a comfortable environment. All interviews were video recorded and transcribed, with participants given the opportunity to review the transcripts for accuracy (Member Check) to avoid bias.

### Data Compilation:

To control biases, the researchers preserved reflective logs after each interview documenting their perceptions about the mentorship program. These reflective logs were discussed with the supervisors and research team to maintain objectivity and minimize personal biases. involved triangulation, with IDIs, FGDs. All the interviews were transcribed and confirmed by members (Member Check).^9^ Data collected from different study groups (leadership, administrators, mentors, and mentees) were triangulated among themselves. Additionally, investigator triangulation was conducted to enhance the validity, credibility, and rigor of the study, where the results from the researcher, investigators, and committee members were compared and analyzed simultaneously.

### Statistical Analysis:

**Fig.1:**
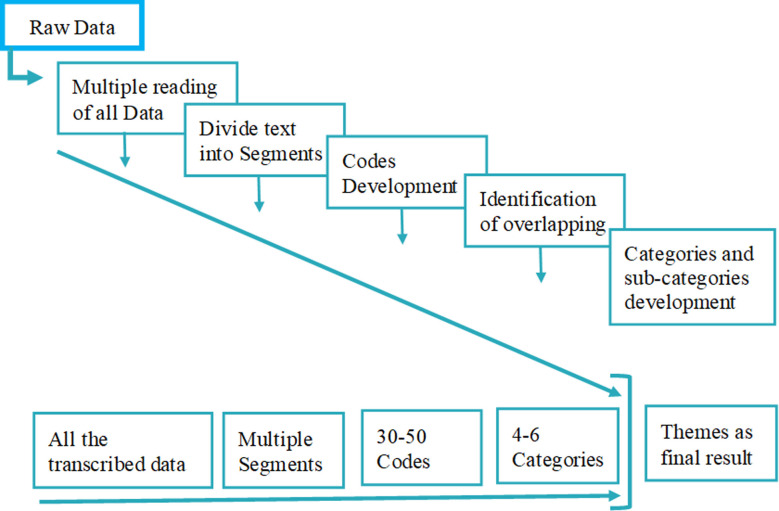
The data analysis flow chart adopted from Creswell (1).

Data was first analyzed manually after which NVivo software was used for schematic representation. For data analysis, the authors independently developed codes to ensure trustworthiness. These codes were then discussed with the committee members. Like codes were merged, grouped into sub-categories and categories, into respective themes,^10^ as given in Fig.1

## RESULTS

Two mentors (females) with 14-20 years of service and two mentees (male) with one to four years of service with AKU-SONAM participated in the study. Dean of the entity, chair and co-chair of the committee who had 20-35 years of involvement in the institution pals participated in the study. The study identified three overarching themes across various categories.

### Theme-1: “Nurturing growth and development in mentoring relationships”:

Discusses the ongoing evolution and advancement of the formal mentorship program at AKU-SONAM. It is delineated by four distinct categories highlighted in Table-I. The progress was evident in the mentees’ development, as articulated by their own accounts. One administrator shared, “*The seed of mentoring at the School of Nursing was planted long before 2015, and significant work has been accomplished in the past two years. We are sufficiently motivated to soon establish a mentorship unit at AKU-SONAM. Another step forward is the introduction of the mentorship award, which complements our ongoing efforts*”.

**Table-I:** Representation of Thematic Analysis AKU-SONAM.

Theme 1:	Category 1:	Category 2:	Category 3:	Category 4:
Nurturing growth and development in mentoring relationships	1. Evolution towards structured mentorship arrangements. 2.Advancement of structured mentoring initiative 3. Mentorship interchange initiative.	1.Assessment of factors for enhanced results 2. structured feedback mechanism 3. Framework for identifying desired outcomes.	1. Role models inspire to recognize potential for excellence. 2. Mentors as exemplars of guidance and inspiration.	1. The most valuable peers are those who elevate you. 2. Assistance from social networks al support
**Theme 2:**	**Category 1:**	**Category 2:**		
Establishing a robust basis for effective mentoring	1. Establishing a clear mentorship trajectory 2. Selection of mentor 3. Responsibilities of the mentors 4. Expectations and obligations of mentees 5. Providing mentorship to more experienced individuals 6. Collaborative efforts across departments	1.Maintaining quality standards 2. Matching mentors and mentees 3. Results-oriented approach 4.Mentorship for research and development purposes 5. Outcomes for knowledge sharing		
**Theme 3:**	**Category 1:**	**Category 2**:		
Opportunities for growth and development through overcoming challenges.	1. Inevitability of challenges and hardships 2. Communication barriers 3. Inefficient time management 4. Insufficient dedication 5. Inadequate administrative support 6. Mismatched mentor-mentee pairs 7. Nonexistence of Framework for Identifying Outcomes	1.Multiple benefits stemming from disciplined efforts 2. Acknowledgment and appreciation of accomplishments 3. Motivation and incentivization 4.Enhancing capabilities and skills		

All the participants highlighted the ‘Importance of Establishing a Formal Feedback System’: This category underscored the necessity of implementing a structured feedback and evaluation system. As expressed by an administrator, “*Currently, performance in clinicals and academics is assessed in a semi-structured manner. To have a structured feedback system, we need written guidelines, a clear flow, and defined processes”*.

### Theme-2: “Establishing a robust basis for effective mentoring”:

Comprised of categories; ‘Establishing a clear mentorship trajectory’ and ‘Maintaining quality standards. Focus of this theme was on the significance of selection processes for novice faculty, the importance of matching mentors with mentees, and the role of administrative support. The mentors shared information about the selection process, chain of command, and workforce with the mentees. An administrator pointed about the selection process as; “*The mentorship committee at SONAM has created a pool of faculty members who have agreed to serve as mentors. The administrators have a list of mentors available for the mentees to choose from based on their specialty, such as research, teaching, or clinical work. Once onboard, the new faculty members have a probationary period of four to six weeks to establish a rapport with their mentor, learn the necessary details, and familiarize themselves with the policies.”* The mentors appreciated the support and opportunities provided by the mentorship program during the selection process.

The mentees hold sole responsibility for initiating steps to achieve their own goals. A mentor emphasized the responsibilities of the mentee by stating, “*Since the mentee gains the most benefits from this partnership in terms of growth and learning, they should take responsibility for being proactive, keeping up to date, and being productive to make this relationship a ’win-win situation”*. A mentor also added; “I *have been mentoring for years and I believe that mentees should take ownership of their learning and growth. Although the process involves collaboration, it is ultimately the mentee who needs support and guidance for their progress and career development. I encourage mentees to take initiative, seek guidance, and support.”*

Although mentoring senior faculty members was seen as a challenge, participants strongly supported its implementation in the faculty mentorship program. ‘Inevitability of challenges and hardships’ and ‘Multiple benefits stemming from disciplined efforts’ constituted.

### Theme-3: “Opportunities for growth and development through overcoming challenges”:

This theme addresses the obstacles and challenges encountered by mentors and mentees throughout their journey, which explains all the barriers and challenges under different subcategories (Table-I). ‘Nonexistence of Framework for Identifying Outcomes’ elucidates the challenge of measuring outcomes in terms of specific parameters due to non-availability of clear guidelines. A mentor voiced their experience, stating, “*Without defined outcome measures, I found it challenging to evaluate the mentee’s progress after a probation period of four to six weeks. We lack a formal tool to assess the learning process or gauge individual mentees’ progress”*.

Administrators and mentors suggested that financial assistance should be provided alongside appreciation letters to further incentivize excellent performance. Additionally, acknowledging mentorship achievements through social media serves as an additional source of motivation, encouragement of faculty for increase in professional development, academic productivity, retention rates hence increasing institutional sustainability. A mentor shared their perspective, stating, “*Appreciation can take various forms such as opportunities for higher education, performance appraisals, participation in international workshops, leadership positions, and awards for mentorship. It is also important to encourage the dissemination of successful mentorship accomplishments through departmental and university newsletters*”.

## DISCUSSION

Our study highlights the importance of academic mentorship with a lasting and supportive connection between seasoned mentors and mentees in AKU-SONAM. All the participants highlighted the scholarly endeavors and developed abilities to navigate the evolving challenges. The stakeholders; administrators, mentors, and mentees agreed that program took considerable time in the development phase and flourished growth of mentors, and mentees in personal advancement, professional development and enhanced competence. The significance of the faculty mentorship program for career guidance, growth, and upgrading in work performance has also been acknowledged by both mentors and mentees at Aga Khan University Medical College.^11^

With reference to “*Nurturing growth and development in mentoring relationships”*, mentors acknowledged that mentorship is a collaborative process, where both the mentors have to provide support and encouragement to mentees. In a national study on student’s mentorship the role of constructive feedback to improve metness confidence was highlighted.^12^ Similarly, expectation of practical and realistic guidance within time frames was also expected from the mentors in doctoral nursing students mentoring program.^13^ Role of faculty mentors has been documented to excel in scholarly output, professional advancement, achieving work-life balance, and integrating into the academic community in nurse health community.^14^

The theme; “*Establishing a robust basis for effective mentoring*” emphasizes on the importance of mentor-mentee matching process, conducting mentoring meetings, monitoring and evaluating the mentoring process, and providing feedback and encouraging reflection in nursing. The participants believed that goals, time lines and key practices should be aimed at building initial rapport.^15^ The description of theme “*Opportunities for growth and development through overcoming challenges”* echoes with the role of organizational support especially for the mentees to effectively navigate the complexities and ever-changing clinical practice environment with regular communication, feedback and support.^2^,^6^ The in-depth exploration in our study corroborates with the role of mentors to support student learning and development in clinical practice, socialization, and improved job performance in the literature.^16^ Within the mentorship program, the mentors and mentees discussed their common interests, expectations, and goals. However, communication gap between mentors and mentees arising from differences in hierarchy, age, experience, and work domains was reported as one of the challenges.^17^ The mentees expressed hesitancy and discomfort in sharing their issues and concerns with their mentors, while mentors expected the mentees to initiate such conversations. Previous research also suggests that formal meetings and workshops can help to address communication gaps and mentoring programs can help in psychological development of individuals.^18^,^19^ Therefore long-term consequences of implementation of mentorship programs can improve the academic performance of metness and provide support to faculty development in all mentoring programs.^20^

### Limitations:

Study’s short duration posed a challenge to evaluate the program’s immediate, intermediate, and long-term outcomes. Nevertheless, the study offered preliminary findings regarding the mentoring program’s efficacy and pinpointed out areas that need attention and require enhancements.

## CONCLUSION

The participants expressed confidence in the existing support system and leadership, recognizing their vital roles in their professional growth. A supportive atmosphere in mentoring relationships was established at AKU-SONAM which nevertheless required further ownership from all stake holders with collaboration, collegiality, reciprocity, frequent communication, feedback, clearly defined objectives, and a commitment to ongoing learning.

### Recommendations

We recommend to introduce ‘Mentee empowerment training sessions’ and ‘Functional mentoring’ by workshops/seminars for both mentors and mentees at AKU and disseminate the program to other universities.
